# Recruitment Challenges and Strategies in a Technology-Based Intervention for Dementia Caregivers: Descriptive Study

**DOI:** 10.2196/59291

**Published:** 2025-01-17

**Authors:** Eunjung Ko, Ye Gao, Peng Wang, Lahiru Wijayasingha, Kathy D Wright, Kristina C Gordon, Hongning Wang, John A Stankovic, Karen M Rose

**Affiliations:** 1 Rory Meyers College of Nursing, New York University, New York, NY, United States; 2 Computer Science, William & Mary, Williamsburg, VA, United States; 3 School of Engineering and Applied Science, University of Virginia, Charlottesville, VA, United States; 4 College of Nursing, The Ohio State University, Columbus, OH, United States; 5 College of Education, Health, and Human Sciences, The University of Tennessee-Knoxville, Knoxville, TN, United States

**Keywords:** recruitment challenges and strategies, technology-based intervention, dementia caregivers, dementia, mobile phone, Alzheimer disease, smart health

## Abstract

**Background:**

Researchers have encountered challenges in recruiting unpaid caregivers of people living with Alzheimer disease and related dementias for intervention studies. However, little is known about the reasons for nonparticipation in in-home smart health interventions in community-based settings.

**Objective:**

This study aimed to (1) assess recruitment rates in a smart health technology intervention for caregivers of people living with Alzheimer disease and related dementias and reasons for nonparticipation among them and (2) discuss lessons learned from recruitment challenges and strategies to improve recruitment.

**Methods:**

The smart health intervention was a 4-month, single-arm trial designed to evaluate an in-home, technology-based intervention that monitors stressful moments for caregiving dyads through acoustic signals and to provide the caregivers with real-time stress management strategies. The recruitment involved two main methods: on-site engagement by a recruiter from a memory clinic and social media advertising. Caregivers were screened for eligibility by phone between January 2021 and September 2023. The recruitment rates, reasons for nonparticipation, and participant demographics were analyzed using descriptive statistics.

**Results:**

Of 201 caregivers contacted, 11 were enrolled in this study. Eighty-two caregivers did not return the screening call, and others did not participate due to privacy concerns (n=30), lack of interest (n=29), and burdensome study procedures (n=26). Our recruitment strategies included addressing privacy concerns, visualizing collected data through a dashboard, boosting social media presence, increasing the recruitment budget, updating advertisements, and preparing and deploying additional study devices.

**Conclusions:**

This study highlighted barriers to participation in the smart health intervention. Despite several recruitment strategies, enrollment rates remained below expectations. These findings underscore the need for future research to explore alternative methods for increasing the recruitment of informal dementia caregivers in technology-based intervention studies.

## Introduction

The number of people living with Alzheimer disease and related dementias (ADRD) in the United States is increasing, with the figure expected to reach 13.8 million by 2060 [1]. Alzheimer disease, the primary neurodegenerative condition contributing to ADRD, necessitates ongoing and escalating levels of support over time for those affected [1]. Most people living with ADRD receive unpaid assistance from family members, friends, or neighbors. In 2023, the number of unpaid caregivers surpassed 11 million [1]. Managing the wide range of caregiving responsibilities and coordinating dementia care can exacerbate caregivers’ stress, burden, anxiety, and depression [1,2].

Various nonpharmacological interventions have emerged to mitigate the negative aspects of caregiving, aimed at supporting caregivers [3]. These interventions include acceptance and commitment therapy, education, counseling, psychotherapy, and support groups. Although the effects of these interventions vary depending on their contexts, the interventions have been generally effective in alleviating caregivers’ negative emotions and improving their quality of life [1,3].

Over the past few decades, technological advancements and the recent pandemic challenges prompted a transition from in-person to online delivery of interventions. This shift has made interventions more accessible by accommodating caregivers’ limited time and geographical locations and alleviating care demand [1,4,5]. The delivery methods of interventions range from the basic tools, such as telephone calls, video conferences, and web page visits, to more advanced technologies such as wearable devices, video or image sensors, or intelligent audio systems [4,6]. These technologies can enhance home safety and well-being and reduce health care costs for people living with dementia and their caregivers [6].

Despite the potential of these novel interventions, their deployment still faces challenges among caregivers of people living with ADRD. Barriers include limited experience with technology, lack of awareness, technical issues, time constraints, privacy concerns, and mistrust [6-8]. Notably, Wen et al [7] found that online-based interventions are more likely to be accepted by younger family caregivers, contrasting with the predominantly middle-aged and older caregiver population [1,9]. Further, the American Association of Retired Persons report revealed that only half of the caregivers across all age groups and just 20% of those older than 65 years of age felt comfortable using in-home technologies (eg, Google Assistant or Alexa; Amazon.com, Inc.) [10]. Given the growing proportion of caregivers aged 60 years and older (28% in 2015 and 35.4% in 2022) [11], participation in technology-based interventions, specifically through those requiring advanced technological devices, remain limited.

Recent studies have explored recruitment challenges and strategies in interventions targeting caregivers of people living with ADRD [7,12-15]. However, there is still limited research specifically focused on these issues within the context of technology-based interventions. Understanding and addressing these challenges are pivotal for effectively engaging caregivers of people living with ADRD in such interventions, which is the primary focus of our study.

In this descriptive study, we aimed to explore the recruitment challenges encountered in an in-home, technology-based intervention designed to reduce stress among caregivers of people living with ADRD and to improve their dyadic relationships. We identified the overall recruitment rate and the reasons for nonparticipation from the caregivers’ perspective and examined the demographic characteristics of the caregivers enrolled in this study. In addition, we discussed the recruitment challenges and strategies we used to address them during the intervention.

## Methods

### Study Design of the Smart Health Intervention

The smart health intervention was a 4-month, single-arm trial designed to develop an in-home, technology-based intervention and assess its feasibility for caregivers of people living with ADRD [16,17]. Caregivers received this study’s devices (ie, a laptop, a microphone connected to the computer, a router, and a smartphone). They placed them in an area of their home where they frequently interacted with their care recipients. We captured acoustic signals from the caregiving dyads through the microphone and used a deep machine-learning approach to identify stressful situations [16]. The system then sent a real-time stress management tip to the caregivers’ study smartphone via the ecological momentary assessment (EMA) system [16,17]. Examples of tips sent to caregivers included practicing body scan and deep-breathing exercises, taking a time out, or engaging in enjoyable activities. The messages encouraged caregivers to follow the tip and to report its usefulness. Caregivers were also asked to answer daily and weekly questions about their physical and emotional health status and the overall usefulness of the stress management tips [17]. We provided US $50 compensation to each caregiver and care recipient three times—1 month after starting the intervention, halfway through, and at the end of the intervention.

### Recruitment

Recruitment took place between January 2021 and September 2023 using convenience sampling. Two main recruitment methods were used to recruit eligible participants: (1) on-site engagement at a memory clinic within a Midwestern academic medical center and (2) advertisements on social media platforms. At the memory clinic, a recruiter distributed study brochures to individuals who visited the clinic and might be eligible for this intervention. Once the caregivers expressed interest in this study, the recruiter shared their contact information with this study’s personnel (EK and KMR). This study’s personnel contacted the caregivers to explain this study’s procedures in detail and confirm their eligibility.

We also advertised the smart health intervention via social media platforms, such as Facebook (Meta) and Instagram (Instagram from Meta), to recruit caregivers across the United States. We allocated between US $100 and US $300 to recruit participants through social media platforms for 1 week of recruitment postings. The uploaded posts included this study’s title, primary text, target age and gender, study locations, and a link to a website containing this study’s detailed information. Individuals who saw this study’s advertisements and were interested in this intervention contacted the principal investigator (KMR) by phone or email. In addition to these two methods, individuals also contacted the principal investigator after finding the information about our study on websites listing clinical trials, such as ClinicalTrials.gov [18].

### Participant Eligibility

We recruited participants who met the following inclusion criteria: (1) aged 21 years or older, (2) residing with and caring for older adults living with ADRD, (3) not receiving payment for providing care, (4) having well-functioning home Wi-Fi, (5) receiving a score above 3 on the Revised Memory and Behavior Problems Checklist, (6) being fluent in English, and (7) agreeing to participate [17]. During the screening call, those who met the eligibility criteria provided verbal consent and signed the electronic consent form. Given that this intervention involved monitoring acoustic signals while caregivers communicated with their care recipients, care recipients were also required to sign the consent form. If care recipients could not consent, their caregivers provided proxy consent.

### Data Collection

#### Overview

We manually recorded the participation status and the reasons for nonparticipation of each individual contacted in REDCap (Research Electronic Data Capture; Vanderbilt University), a Health Insurance Portability and Accountability Act compliant, web-based data collection platform that allows researchers to access and manage data [19]. We also gathered the reasons for nonparticipation during the screening call. After individuals agreed to participate, we emailed them a REDCap link to the consent form and the demographic questionnaire. Those who consented and completed the questionnaires received copies of the informed consent form. Participants were required to complete the survey only through this link, with responses automatically stored in REDCap.

#### Participation Status and Reasons for Nonparticipation

During the screening call, we asked individuals screening questions and documented their reasons for nonparticipation. The reasons were categorized into predeveloped options: (1) did not meet eligibility criteria, (2) not interested, (3) considered study protocol to be too burdensome, (4) worried about privacy issues, (5) others (eg, considering care replacement to facilities), (6) expressed interest but did not respond to the screening call, (7) noted invasion of privacy was undesirable, (8) harmful events occurred due to this study, and (9) no longer wanted to participate.

#### Demographic Characteristics of Participants

We collected demographic data from participants at baseline. The questionnaire included information on the age, gender, race (American Indian or Alaska Native, Asian Native Hawaiian or other Pacific Islander, Black or African American, White, unknown, or not reported), ethnicity (Hispanic or Latino, Not Hispanic or Latino, or not reported), relationship between caregivers and care recipients (spouse, partner, sibling, child, grandchild, or other), education level (never attended or kindergarten only, K grades from 1st to 12th [not diploma], high school graduate, general education diploma or equivalent, attended college but did not obtain a degree, associate degree, bachelor’s degree, master’s degree, professional school, doctoral degree, or unknown), employment status (working now, only temporarily laid off, sick leave, maternity leave, unemployed, retired, disabled permanently or temporarily, keeping house, student, other, or unknown), caregiving hours per day, caregiving duration in months, marital status, the number of household members, zip code, veteran status (yes or no), and any history of combat exposure for those with a military background.

#### Challenges and Strategies in Recruitment

To address recruitment challenges through on-site engagement, we conducted two 20-minute Zoom (Zoom Communications, Qumu Corporation) meetings with a memory clinic recruiter in June and November 2022. We also held weekly meetings with the research team to discuss challenges in recruiting through social media and brainstorm potential solutions.

### Ethical Considerations

The smart health intervention was approved by the institutional review board at this study’s institution (2019B0406) and was registered with ClinicalTrials.gov (NCT04536701) on September 3, 2020, and completed on December 31, 2023. All participants provided written, informed consent before the data collection. Participants were informed about this study’s purpose, duration, location of data storage, potential benefits and risks of involvement in the intervention, and contact information for the principal investigators.

### Data Analysis

We used SPSS (version 29; IBM Corp) for descriptive analyses, including frequencies, percentages, means, and SDs, to present the characteristics of recruitment and participant demographics. We also used a note-taking approach during discussions with a recruiter and team members to capture key points on the topic.

## Results

### Participation Status and Reasons for Nonparticipation

**Figure 1.**
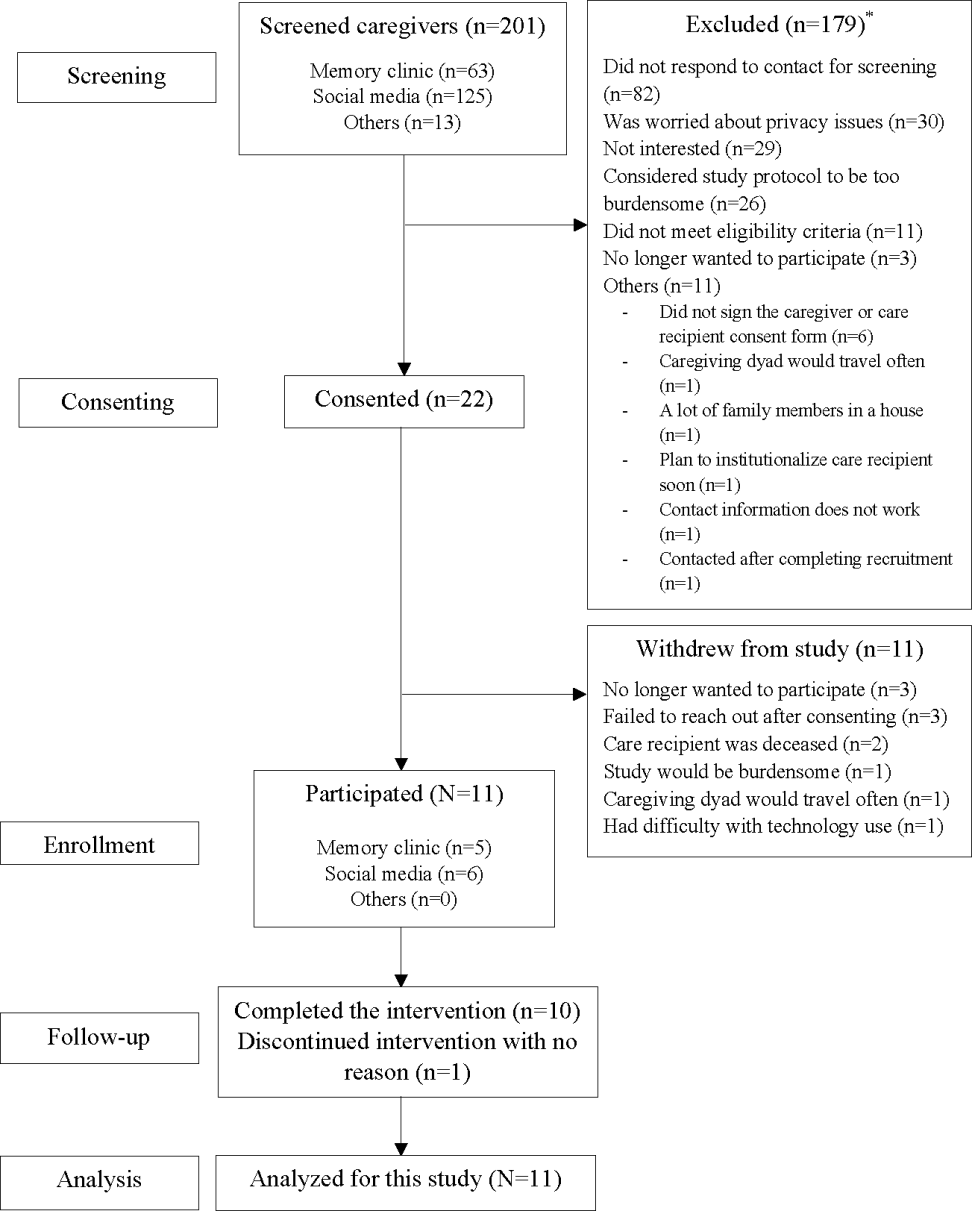
Flowchart illustrating participant recruitment and exclusion. *Some of 179 respondents addressed multiple reasons for their non-participation.

Figure 1 presents the recruitment flowchart and reasons for nonparticipation. We attempted to screen 201 caregivers through social media (125 individuals), memory clinic (63 individuals), and other sources, such as ClinicalTrials.gov or unknown routes (13 individuals). Of these, 22 caregivers and their care recipients agreed to consent; only half completed the baseline questionnaire and began the intervention. Six of the 11 participants were recruited through social media, and the remaining 5 were recruited through on-site engagement at the memory care clinic (Figure 1).

Among caregivers excluded, 82 did not respond to the screening contact, 30 declined to participate due to privacy concerns, 29 were not interested in the intervention, and 26 considered this study’s protocol to be burdensome. Eleven respondents failed to meet the eligibility criteria, mainly because they were not primary or live-in caregivers, their care recipient was hospitalized or deceased, or they lacked functional Wi-Fi at home. Three caregivers expressed interest but later decided not to participate after further consideration. Other reasons for nonparticipation included (1) not providing written consent on a consent form after providing verbal agreement, (2) frequent travel plans, (3) having multiple household members besides the caregiving dyad, (4) planning to institutionalize their care recipient soon, and (5) providing incorrect contact information. Further, 11 caregivers who consented ultimately withdrew from this study for reasons such as no longer wanting to participate, failure to reach out, and the death of the care recipient.

### Demographic Characteristics of Participants

Table 1 presents the demographic data for the participating caregiving dyads. The average ages of caregivers and care recipients were 60.09 (SD 13.52) years and 76.73 (SD 8.04) years, respectively. Most caregivers were female (n=8, 73%), while most care recipients were male (n=7, 64%). Most caregiving dyads were non-Hispanic White (n=7, 82% of caregivers; n=9, 91% of care recipients). Education levels varied, but all caregivers had attained education beyond a high school degree. Regarding employment status, 8 (73%) caregivers and all care recipients were either unemployed or retired. Eight (73%) care recipients and 7 (64%) caregivers were married, with caregivers typically being spouses. The average amount of caregiving hours per day was 21.18 (SD 6.37) hours, and the average caregiving duration was 34.82 (SD 20.26) months.

**Table 1.** Demographics of actual participating dyads (N=11).

Characteristic	Caregivers	Care recipients
Age (years), mean (SD)	60.09 (13.52)	76.73 (8.04)
**Gender, n (%)**		
Female	8 (73)	4 (36)
Male	3 (27)	7 (64)
**Race, n (%)**		
African American or Black	1 (9)	1 (9)
Asian American	1 (9)	0 (0)
Non-Hispanic White	9 (82)	10 (91)
**Ethnicity, n (%)**		
Non-Hispanic	10 (91)	10 (91)
Not reported	1 (9)	1 (9)
**Education level, n (%)**		
General education diploma or equivalent	0 (0)	1 (9)
12th grade, not diploma	0 (0)	1 (9)
High school graduate	0 (0)	1 (9)
Attended college but did not obtain a degree	1 (9)	2 (18)
Associate degree	2 (18)	2 (18)
Bachelor’s degree	4 (36)	1 (9)
Master’s degree	3 (27)	1 (9)
Doctoral degree	1 (9)	2 (18)
**Employment status, n (%)**		
Employed	3 (27)	0 (0)
Not employed or retired	8 (73)	11 (100)
**Marital status, n (%)**		
Married	7 (64)	8 (73)
Never married	3 (27)	0 (0)
Widowed	0 (0)	2 (18)
Divorced	1 (9)	1 (9)
**Veteran status and combat exposure, n (%)**		
Yes, no combat exposure	2 (18)	1 (9)
No	9 (82)	10 (91)
Caregiving hours (h/d), mean (SD)	21.18 (6.37)	—^a^
Caregiving duration (mo), mean (SD)	34.82 (20.26)	—
Household member, mean (SD)	2.27 (0.65)	—

a The “—” indicates “not applicable”

### Lessons Learned Through Recruitment Challenges

#### Challenges of On-Site Engagement

A recruiter responsible for informing caregivers about this study at the memory clinic provided some reasons for a low rate of face-to-face recruitment. First, staffing shortages at the clinic and the shift from in-person to remote medical appointments likely reduced recruitment opportunities. When patients transitioned to telehealth appointments, the recruiter could not meet with them and their caregivers to provide study information. Additionally, increased patient no-shows for in-person medical appointments further hindered recruitment efforts. Caregivers also expressed concerns about the time commitment and privacy issues associated with technology-based interventions, which echoed the feedback from the caregivers who refused participation in the smart health intervention. The recruiter noted that caregivers showed more interest in pharmacological trials for their care recipients than in nonpharmacological trials for themselves.

To address these challenges, the recruiter suggested introducing this study virtually before or after telehealth appointments to raise awareness among caregivers and their care recipients. Increasing the clinic’s staff who help study recruitment and having study personnel provide detailed explanations of this study—either in person or virtually—while caregivers wait for their care recipients could also help improve recruitment rates.

#### Challenges of Social Media Recruitment

We also encountered specific challenges with recruiting through social media platforms. A primary issue was the need to present study information briefly within social media posts, which often limited the detail we could provide. This brevity hindered potential participants from receiving detailed study information to decide, which is particularly critical in intervention studies. For example, of 601 caregivers who clicked the advertisements in April, only 7 reached out to the staff for further information. This suggests that the brief content in the advertisements may not have efficiently delivered this study’s purpose or importance to potential participants.

Another issue was that the visibility of our study was directly tied to our advertising budget. As a result, we had to allocate more resources to increase our reach on social media platforms. Moreover, we faced difficulties maintaining the balance between demand and supply. When study advertisements were posted on social media, we received a flood of inquiries from interested participants, but limitations in the availability of study equipment caused delays. Some participants had to wait weeks or months to receive this study’s equipment, likely contributing to decreased interest and higher withdrawal rates.

#### Strategies to Improve Recruitment

We discussed and attempted several strategies to alleviate the recruitment challenges, specifically targeting the individual concerns that led to the reluctance to participate. To mitigate privacy concerns in the smart health intervention, we reassured caregivers during recruitment that we would not record their conversation or behaviors but focus on the changes in acoustic characteristics, such as tone, intonation, and amplitude. We allowed participants to choose the microphone placement for data collection, provided it was in a room where frequent interactions with the care recipient occurred. Additionally, we clarified that we would only analyze acoustic data collected from the participating caregiving dyad, differentiating their voices from others in the household (ie, visitors).

**Figure 2.**
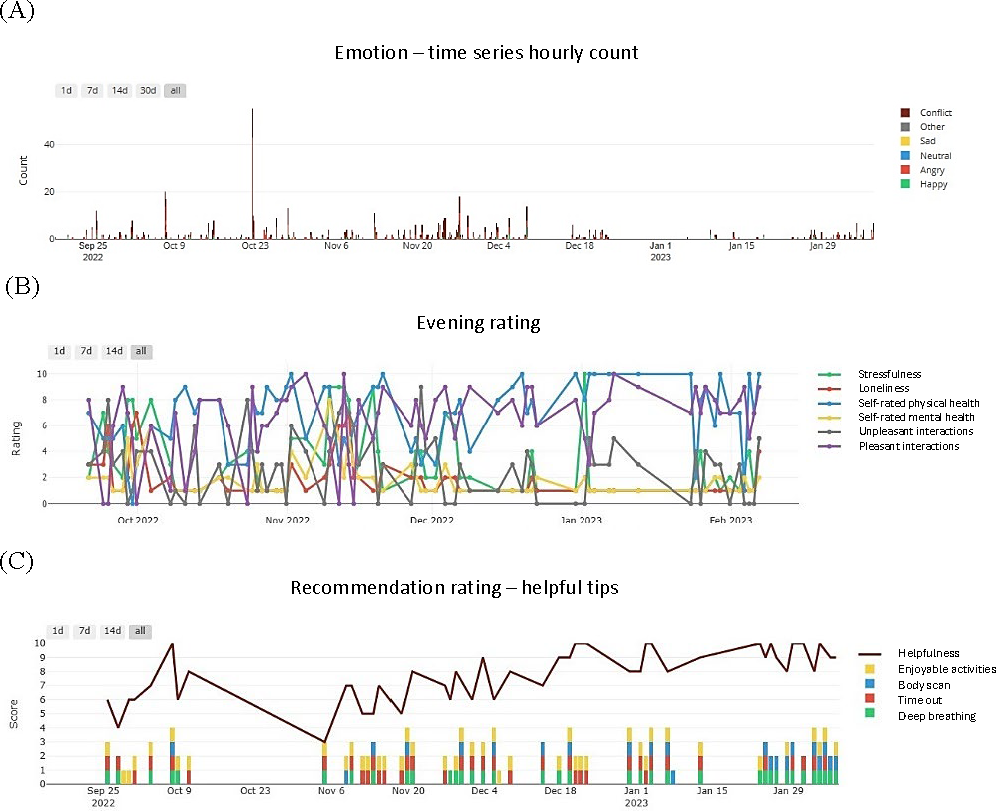
Examples of dashboard data collection. These graphs were generated based on an actual participant’s data. The x-axis represents the passage of time. (A) The cumulated number of daily mood states through acoustic monitoring. (B) Self-reported daily emotional status. (C) Followed recommendation tips per day (bars) and daily scores for helpfulness (line).

We also developed a dashboard (Figure 2) and shared it with participants, showing the visualized data without capturing specific words or conversations. This approach helped ease privacy concerns, and as this study progressed, participants gained confidence in the system’s utility. By the end of this study, they reported that privacy was no longer a concern.

To enhance recruitment through social media, we consulted with social media specialists on ways to improve the visibility of this study’s advertisements. We increased our budget for social media advertising from US $100/wk in April to US $200/wk in September and revised the advertising text to provide more detailed information for eligible caregivers (Textbox 1). These modifications led to a slight rise in interest from potential study participants, as indicated by an increase in advertisement clicks from 601 in April to 1421 in September. The number of inquiries for detailed information also grew, from 7 in April to 17 in September. However, there was no change in terms of percentage (from 7/601, 1.16% in April to 16/1701, 1.20%), while the cost per click was higher (from US $0.33 in April to US $0.42 in September). Despite these efforts, we recruited only 5 out of 125 participants through social media, indicating that the strategies may not be effective and that further actions are needed to capture the attention of potential participants better.

Textbox 1. **Changes in the statements on advertising posts.**

Statement on the advertisements *before* the revision

Researchers at the ### are seeking participants for a study to learn more about ways we can help family caregivers for persons with memory loss manage their stress better. The research study will help family caregivers recognize their stressful situations and find ways they may be able to feel less stress.

Statement on the advertisements *after* the revision

Researchers at the ### are seeking family or friends who care for people with memory loss for a study. *By identifying voice changes, this study* will help caregivers recognize their feelings in tense moments and *cope with their difficult conversations with persons with memory loss*. This may help caregivers find ways to i*ncrease self-care and satisfaction with their daily lives. You do not have to be good with technology to participate. Participant privacy will be carefully protected. Please do not hesitate to contact us with questions or for more information!*

We prepared and deployed additional study devices for participants on the waitlist. To enhance their motivation to participate in this study, we conducted regular follow-ups, approximately 2 to 3 times monthly. These actions enabled us to monitor and address study-related challenges and facilitate effective communication and rapport between study personnel and participants, helping ensure their continued involvement in this study. However, some potential participants experienced changes in their caregiving situations (eg, planning travel, experiencing a care recipient’s death, or having second thoughts about engaging in this study) and withdrew from the waitlist.

## Discussion

### Principal Findings

This study aimed to identify recruitment challenges in a smart health technology intervention for caregivers of people living with dementia. We observed a low recruitment rate and identified various reasons for nonparticipation among caregivers. Beyond nonresponsiveness to screening, the most common reasons for nonparticipation included privacy concerns, a lack of interest, and perceptions of this study as being burdensome. The demographic characteristics of participating caregivers showed that the majority were mainly female, White, spousal caregivers, and retired. Recruitment challenges were observed both on-site and through social media. On-site engagement challenges included the transition to remote medical appointments and staffing issues. At the same time, social media-based recruitment faced limitations related to the context of postings, budget considerations, and balancing demand and supply.

We observed a lower recruitment rate through social media (6/125 individuals) than on-site engagement (5/63 individuals). Previous literature has reported mixed results regarding the effectiveness of using social media for recruitment in intervention research [13,20-26]. While some studies have suggested that social media recruitment could be costly and ineffective [20-22,24], others have argued that social media could benefit the recruitment of potential target participants [13,23,25,26]. These conflicting findings highlight a need for further research to explore differences in recruitment rates across recruitment channels in technology-based interventions for people living with ADRD and their caregivers.

Caregivers of people living with ADRD may be anxious about sharing their data to use technology [27-29]. Additionally, they often prioritize dementia care and pay more attention to clinical trials focused on treating a specific disease than trials targeting their health [30]. While multiple pharmacological clinical trials may be ongoing in a clinic, caregivers of people living with ADRD may overlook nonpharmacological intervention studies. Moreover, they already spend considerable time and energy caring for their care recipients, and they may perceive participating in an intervention study, especially a technology-based trial, to be overly demanding [29,31,32]. Therefore, intervention research using technology should use strategies that reduce the perceived burden, such as providing training, using user-friendly platforms, or offering nondigital alternatives, including daily logs for those uncomfortable with technology in the caregiving population [8]. These tailored approaches have the potential to enhance the enrollment in dementia caregiving research.

Being female and non-Hispanic White are common characteristics of caregivers of people living with dementia in the United States [1], and spousal dementia caregivers are more likely to be retired [33]. Compared to male caregivers, females are more likely to sacrifice personal time, which can lead to a more significant burden and depressive symptoms [1]. Spousal dementia caregivers are also at a higher risk for depression, with rates being 2.5 times higher than that for nonspousal caregivers [1]. Caregivers with these characteristics may be more willing to participate in caregiving research on stress management. Still, the lack of demographic data on nonparticipants makes it difficult to confirm this. Therefore, future studies should gather information from participants and nonparticipants to better understand why some caregivers choose not to engage in research.

### Limitations and Potential Implications

Despite our efforts, the strategies implemented in the smart health intervention needed to address recruitment barriers effectively. First, we needed more complete data on the socioeconomic status and race or ethnicity of potential participants who did not enroll in our study. Research shows that dementia caregiving dyads from lower socioeconomic backgrounds are less likely to participate in studies involving technology due to unfamiliarity and limited access to technological devices [31,34]. This gap in our data limited our ability to access the potential impact of the backgrounds of caregiving dyads on the recruitment rate.

Second, concerns about this study being too demanding persisted throughout recruitment, likely due to caregivers’ unfamiliarity with the technology. This study’s design, which included real-time messaging through the EMA system, may have contributed to this perception. However, an intervention study using the EMA system reported that the EMA approach did not bother participating caregivers [35]. Nonetheless, more research is needed to understand how the EMA system affects caregivers’ daily lives while capturing data [36].

We could not manage the recruitment challenges, such as the transition to remote medical appointments and staffing issues, through on-site engagement. Recruitment might have improved with more advertising before or after virtual medical appointments, greater research personnel involvement, and additional resources; however, barriers to participating in telehealth visits made implementing these strategies unfeasible. The rise in health care since the COVID-19 pandemic—an approximately 150% increase in telehealth visits [37]—posed a barrier to recruiting caregivers in person. Future studies should explore ways to overcome these challenges when recruiting participants with limited in-person contact.

Despite these limitations, the findings of this study can inform strategies for addressing recruitment challenges in dementia caregiving research. Our findings offer insights into the reasons for nonparticipation and how to address participants’ concerns regarding the smart health intervention. The findings may help researchers develop better strategies to improve recruitment rates in similar studies, offering caregivers opportunities to enhance their health and the quality of care they provide to their loved ones.

## Abbreviations

ADRD: Alzheimer disease and related dementias
EMA: ecological momentary assessment
REDCap: Research Electronic Data Capture

## References

[1] (2024). 2024 Alzheimer’s disease facts and figures. Alzheimers Dement.

[2] Pinyopornpanish K, Soontornpun A, Wongpakaran T (2022). Impact of behavioral and psychological symptoms of Alzheimer’s disease on caregiver outcomes. Sci Rep.

[3] Sun Y, Ji M, Leng M, Li X, Zhang X, Wang Z (2022). Comparative efficacy of 11 non-pharmacological interventions on depression, anxiety, quality of life, and caregiver burden for informal caregivers of people with dementia: a systematic review and network meta-analysis. Int J Nurs Stud.

[4] González-Fraile E, Ballesteros J, Rueda JR, Santos-Zorrozúa B, Solà I, McCleery J (2021). Remotely delivered information, training and support for informal caregivers of people with dementia. Cochrane Database Syst Rev.

[5] Sharma RK, Teng A, Asirot MG, Taylor JO, Borson S, Turner AM (2022). Challenges and opportunities in conducting research with older adults with dementia during COVID-19 and beyond. J Am Geriatr Soc.

[6] Ali MT, Turetta C, Demrozi F, Pravadelli G (2024). ICT-based solutions for Alzheimer’s disease care: a systematic review. IEEE Access.

[7] Wen Y, Xing Y, Ding Y, Xu W, Wang X (2023). Challenges of conducting of online educational programs for family caregivers of people with dementia living at home: an integrative review. Int J Nurs Sci.

[8] Brookman R, Parker S, Hoon L (2023). Technology for dementia care: what would good technology look like and do, from carers’ perspectives?. BMC Geriatr.

[9] Brodaty H, Donkin M (2009). Family caregivers of people with dementia. Dialogues Clin Neurosci.

[10] Keenan TA (2022). U.S. caregivers’ use of technology.

[11] Kilmer G, Omura JD, Bouldin ED (2024). Changes in health indicators among caregivers - United States, 2015-2016 to 2021-2022. MMWR Morb Mortal Wkly Rep.

[12] Joshi S, Park T, Brody L (2023). Recruitment of family caregivers of persons with dementia: lessons learned from a pilot randomized controlled trial. Front Pain Res (Lausanne).

[13] Baker FA, Blauth L, Bloska J (2023). Recruitment approaches and profiles of consenting family caregivers and people living with dementia: a recruitment study within a trial. Contemp Clin Trials Commun.

[14] Langbaum JB, Zissimopoulos J, Au R (2023). Recommendations to address key recruitment challenges of Alzheimer’s disease clinical trials. Alzheimers Dement.

[15] Yang M, Samper-Ternent R, Volpi E (2024). The dementia care study (D-CARE): recruitment strategies and demographic characteristics of participants in a pragmatic randomized trial of dementia care. Alzheimers Dement.

[16] Gao Y, Jabbour J, Schlegel EC (2021). Out-of-the-box deployment to support research on in-home care of Alzheimer’s patients. IEEE Pervasive Comput.

[17] Rose KM, Gordon KC, Schlegel EC (2021). Smarthealth technology study protocol to improve relationships between older adults with dementia and family caregivers. J Adv Nurs.

[18] Rose K (2024). Collaborative research: learning and improving Alzheimer’s patient-caregiver relationships via smart healthcare technology. Clinicaltrials.gov.

[19] Harris PA, Taylor R, Thielke R, Payne J, Gonzalez N, Conde JG (2009). Research electronic data capture (REDCap)--a metadata-driven methodology and workflow process for providing translational research informatics support. J Biomed Inform.

[20] Beattie E, Fielding E, O’Reilly M, Brooks D, MacAndrew M, McCrow J (2018). Recruitment of individuals with dementia and their carers for social research: lessons learned from nine studies. Res Gerontol Nurs.

[21] Leach MJ, Ziaian T, Francis A, Agnew T (2016). Recruiting dementia caregivers into clinical trials: lessons learnt from the Australian TRANSCENDENT trial. Alzheimer Dis Assoc Disord.

[22] Leslie M, Khayatzadeh-Mahani A, MacKean G (2019). Recruitment of caregivers into health services research: lessons from a user-centred design study. Res Involv Engagem.

[23] Wasfi R, Stephens ZP, Sones M (2021). Recruiting participants for population health intervention research: effectiveness and costs of recruitment methods for a cohort study. J Med Internet Res.

[24] Whitlatch CJ, Orsulic-Jeras S, Johnson J (2021). Challenges to and strategies for recruiting chronic care dyads into intervention research. Chronic Illn.

[25] Bremer W, Sarker A (2023). Recruitment and retention in mobile application-based intervention studies: a critical synopsis of challenges and opportunities. Inform Health Soc Care.

[26] Darko EM, Kleib M, Olson J (2022). Social media use for research participant recruitment: integrative literature review. J Med Internet Res.

[27] Block L, Gilmore-Bykovskyi A, Jolliff A, Mullen S, Werner NE (2020). Exploring dementia family caregivers’ everyday use and appraisal of technological supports. Geriatr Nurs.

[28] Christie HL, Bartels SL, Boots LMM, Tange HJ, Verhey FRJ, de Vugt ME (2018). A systematic review on the implementation of eHealth interventions for informal caregivers of people with dementia. Internet Interv.

[29] Hassan AYI (2020). Challenges and recommendations for the deployment of information and communication technology solutions for informal caregivers: scoping review. JMIR Aging.

[30] Clemmensen TH, Lauridsen HH, Andersen-Ranberg K, Kristensen HK (2021). “I know his needs better than my own” - carers’ support needs when caring for a person with dementia. Scand J Caring Sci.

[31] Brody AA, Convery KA, Kline DM, Fink RM, Fischer SM (2022). Transitioning to remote recruitment and intervention: a tale of two palliative care research studies enrolling underserved populations during COVID-19. J Pain Symptom Manage.

[32] Trivedi RB, Szarka JG, Beaver K (2013). Recruitment and retention rates in behavioral trials involving patients and a support person: a systematic review. Contemp Clin Trials.

[33] Torres JM, Romero KRF, Kotwal AA (2024). Spouses of individuals living with mild cognitive impairment or dementia in the United States: a descriptive, population-based study. Alzheimers Dement.

[34] AboJabel H, Welsch J, Schicktanz S (2024). Cross-cultural perspectives on intelligent assistive technology in dementia care: comparing Israeli and German experts’ attitudes. BMC Med Ethics.

[35] van Knippenberg RJM, de Vugt ME, Ponds RW, Myin-Germeys I, van Twillert B, Verhey FRJ (2017). Dealing with daily challenges in dementia (deal-id study): an experience sampling study to assess caregiver functioning in the flow of daily life. Int J Geriatr Psychiatry.

[36] Han A, Malone LA, Lee HY (2024). The use of ecological momentary assessment for family caregivers of adults with chronic conditions: a systematic review. Health Psychol Res.

[37] Koonin LM, Hoots B, Tsang CA (2020). Trends in the use of telehealth during the emergence of the COVID-19 pandemic - United States, January-March 2020. MMWR.

